# Review of the genus *Fontidessus* Miller & Spangler, 2008 (Coleoptera, Dytiscidae, Hydroporinae, Bidessini) with description of four new species

**DOI:** 10.3897/zookeys.426.7217

**Published:** 2014-07-17

**Authors:** Kelly B. Miller, Elizabeth T. Montano

**Affiliations:** 1 Department of Biology, University of New Mexico, Albuquerque, NM 87131–0001 USA

**Keywords:** Guiana Shield, hygropetric, taxonomy, water beetle

## Abstract

The genus *Fontidessus* Miller & Spangler, 2008 (Coleoptera: Dytiscidae: Hydroporinae: Bidessini) is reviewed. The genus now includes seven species with three previously described, and four new species described here: *F. microphthalmus* Miller & Montano, **sp. n.**; *F. bettae* Miller & Montano, **sp. n.**; *F. christineae* Miller & Montano, **sp. n.**, and *F. aquarupe* Miller & Montano, **sp. n.** Each species is diagnosed and described, including the previously known species, based on new specimens and new information. Habitus, male genitalia and other diagnostic features are illustrated for each species. A key to the seven species is provided. *Fontidessus* species are unique to hygropetric habitats in the Guiana Shield craton of northern South American.

## Abstract (es)

El género *Fontidessus* Miller & Spangler, 2008 (Coleoptera: Dytiscidae: Hydroporinae: Bidessini) es revisado. El género incluye siete especies, con tres descritas anteriormente, y cuatro nuevas especies descritas aquí: *F. microphthalmus* Miller & Montano, **sp. n.**, *F. bettae* Miller & Montano, **sp. n.**, *F. christineae* Miller & Montano, **sp. n.**, y *F. aquarupe* Miller & Montano, **sp. n.** Las especies se diagnostican y se describen, incluyendo las especies previamente conocidas, basado en nuevos ejemplares e información. Habitus, genitales masculinos y otras características diagnósticas se ilustran para cada especie. Se presenta una clave para las siete especies. *Fontidessus* son exclusivos de hábitats hygropétricos en el Cratón de Guayana Escudo del norte de América del Sur.

## Introduction

*Fontidessus* Miller & Spangler was introduced to include three new species of diving beetles (Coleoptera: Dytiscidae) from northern South America (Miller and Spangler 2008). Members of the genus are characteristic of habitats where thin films of water flow over the surfaces of bare rock (hygropetric). Northern South America has a relatively rich fauna of hygropetric beetles including numerous diving beetles (Miller and Garcia 2011; Miller and Spangler 2008) and other water beetles (Miller 2009; Short and Garcia 2010; Spangler and Steiner 2005; Valladares and Short 2011). Most of this known diversity is from the western margin of the Guiana Shield. Additional collecting in Venezuela and farther east in the Guiana Shield region (Guyana and Suriname) and examination of collections in Venezuela has revealed additional new species and new distribution records for the described species. Less is known about hygropetric species in the eastern part of the Shield, and the presence of several new species in this recently described genus is tantalizingly suggestive of additional new diversity as this habitat becomes better explored. Two of the original known species were described from only few specimens, and the new material has clarified their morphology and variation considerably. Specimens of most species of *Fontidessus* are exceptionally small, even for Bidessini, and their male genitalia are complex and tiny with minute, diaphanous structures making critical examination difficult. Because of substantial new information, the entire genus is reviewed here with descriptions of four new species and clarifications of and corrections to the knowledge of distribution, morphology and variation of the three known species.

## Materials and methods

Measurements were taken with an ocular scale on a Leica Discovery V8 dissecting microscope. Large and small specimens were measured to assess the range of sizes, and no attempt was made to determine average sizes. Measurements include: 1) total length (TL), 2) greatest width across elytra (GW), 3) greatest width of pronotum (PW), 4) greatest width of head (HW), and 5) distance between eyes (EW). The ratios TL/GW and HW/EW were also calculated and are provided to describe the overall shape and relative size of the eyes, respectively.

Specimens studied for this project are deposited in the Natural History Museum and Biodiversity Research Center, University of Kansas (SEMC, A.E.Z. Short), the National Zoological Collection of Suriname (NZCS, A. Gangadin), the Museo del Instituto de Zoología Agrícola Francisco Fernández Yépez, Universidad Central de Venezuela, Maracay, Venezuela (MIZA, L. Joly), the Center for Biological Diversity, University of Guyana (CSBD), and the Museum of Southwestern Biology Division of Arthropods, University of New Mexico (MSBA, K.B. Miller). Holotype deposition is indicated in the material examined sections for each species. Paratypes are distributed between these collections.

In the following treatments, species are organized according to general similarity (and their appearance in the key that follows), but it is not clear at this time what are the relationships between species of *Fontidessus*.

## Taxonomy

### Fontidessus

Taxon classificationAnimaliaColeopteraDytiscidae

Miller & Spangler, 2008

Fontidessus Miller & Spangler, 2008: 46.

#### Type species.

*Fontidessus toboganensis* Miller & Spangler, 2008 by original designation.

#### Diagnosis.

**Figures 1–9.**
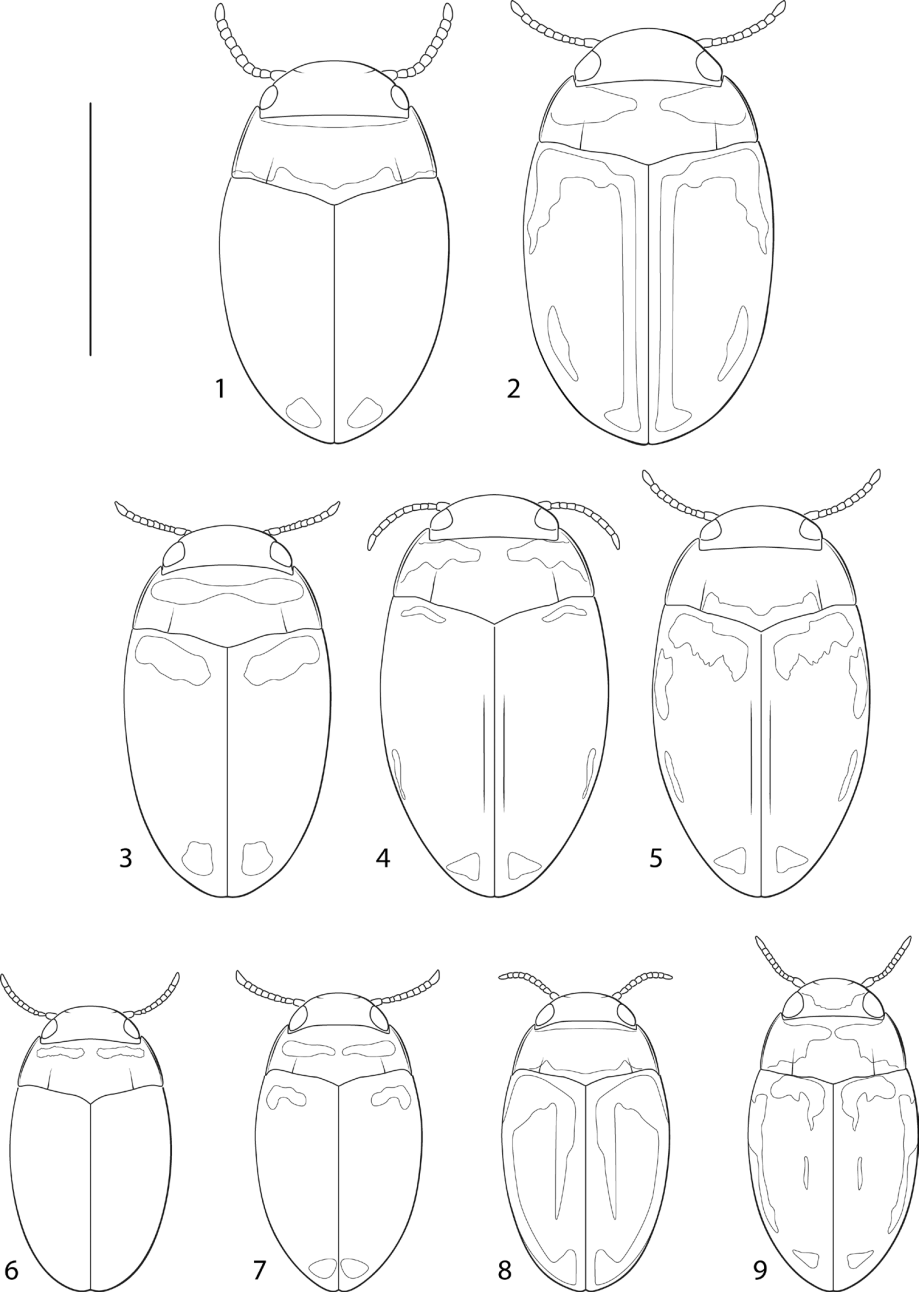
*Fontidessus* species, dorsal habitus. **1** *Fontidessus microphthalmus* **2** *Fontidessus bettae* **3** *Fontidessus bettae* **4–5** *Fontidessus toboganensis* **4** Suriname specimen **5** Venezuela specimen **6** *Fontidessus christineae* **7–8** *Fontidessus ornatus* **7** Suriname specimen **8** Venezuela specimen **9** *Fontidessus wheeleri*. Scale = 1.0 mm.

**Figures 10–23.**
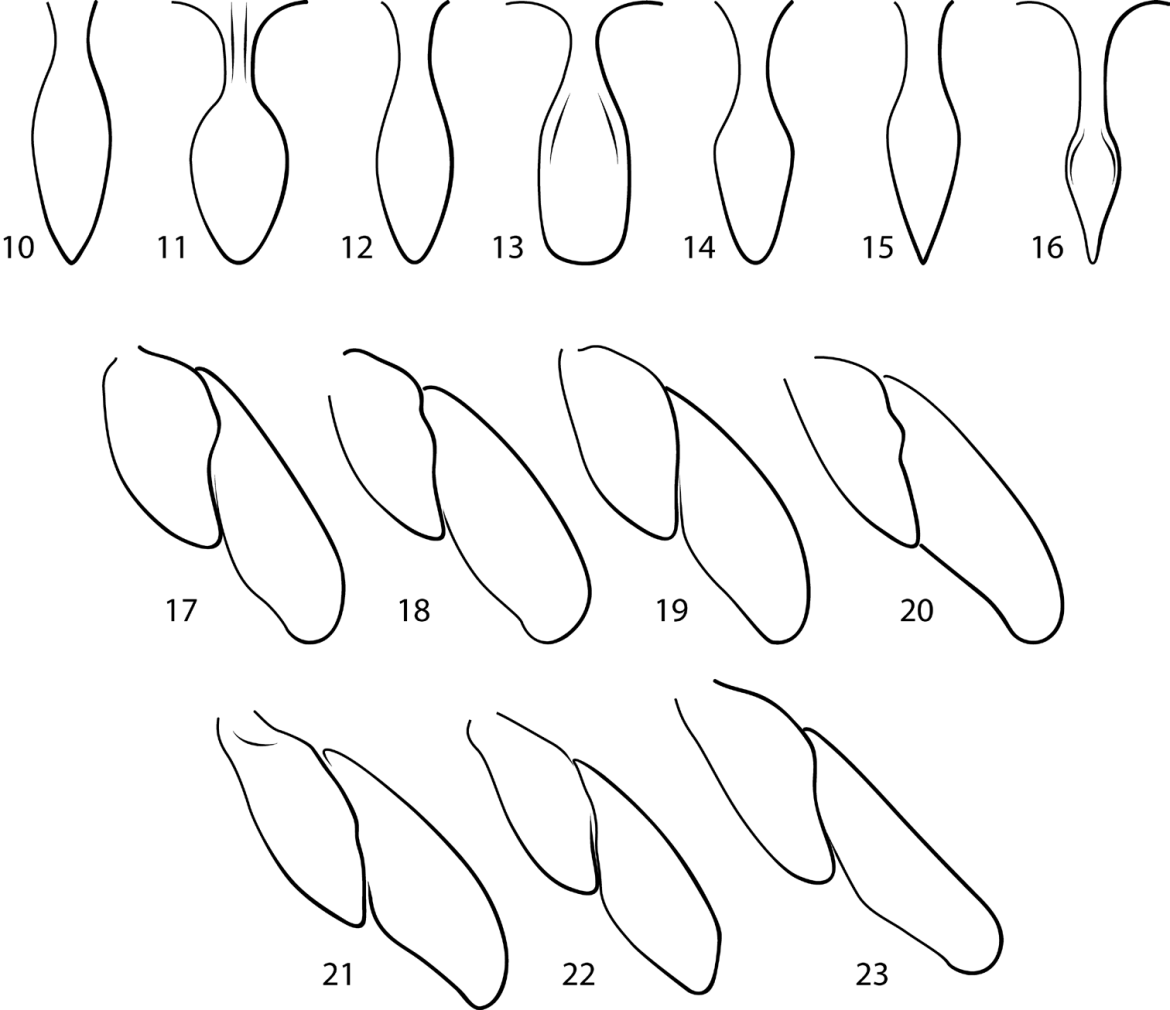
*Fontidessus* species. **10–16** prosternal process **10** *Fontidessus microphthalmus* **11** *Fontidessus aquarupe* **12** *Fontidessus bettae* **13** *Fontidessus toboganensis* **14** *Fontidessus christineae* **15** *Fontidessus ornatus* **16** *Fontidessus wheeleri* **17–23** left metatrochanter and metafemur, anterior aspect **17** *Fontidessus microphthalmus* **18** *Fontidessus bettae* **19** *Fontidessus toboganensis* **20** *Fontidessus christineae* **21** *Fontidessus ornatus*. 22, *Fontidessus wheeleri*.

**Figures 24–30.**
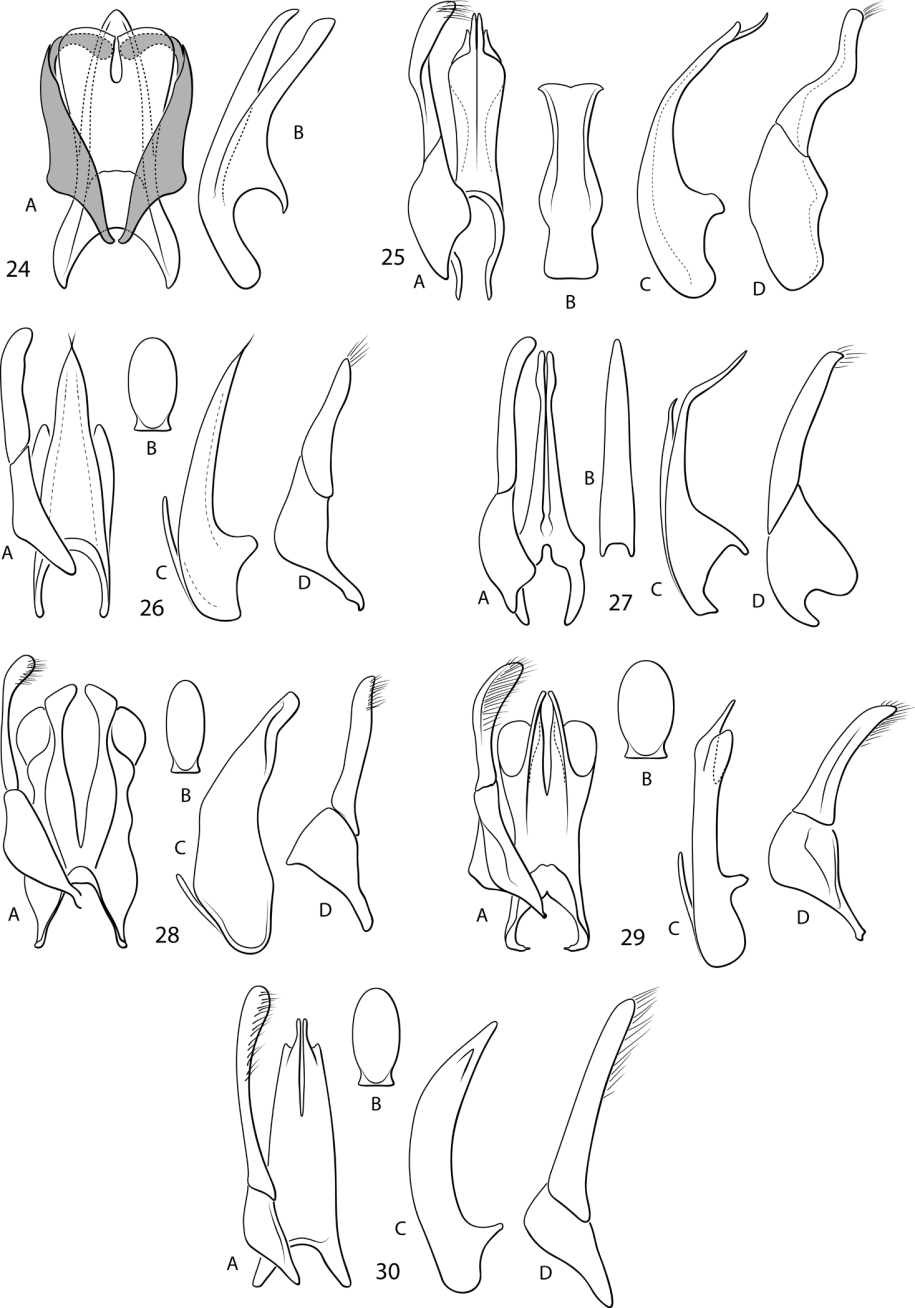
*Fontidessus* species, aedeagi. **24** *Fontidessus microphthalmus*; A, median lobe and lateral lobes, dorsal aspect, lateral lobes in gray; B, median lobe, right lateral aspect **25–30** A, median lobe and right lateral lobe, dorsal aspect; B, ventral sclerite of median lobe; C, median lobe, right lateral aspect; D, right lateral lobe, right lateral aspect **25** *Fontidessus aquarupe* **26** *Fontidessus bettae* **27** *Fontidessus toboganensis* **28** *Fontidessus christineae* **29** *Fontidessus ornatus* **30** *Fontidessus wheeleri*.

*Fontidessus* differs from other Bidessini by the combination of: 1) a transverse occipital line across the head absent (Figs 1–9), 2) a pair of basal pronotal striae present (Figs 1–9), 3) a basal elytral stria absent (Figs 1–9), 4) an elytral sutural stria faintly present in some specimens (e.g. Figs 4, 5), 5) the anterior clypeal margin unmodified, evenly rounded 6) the elytron without longitudinal carinae, 7) the elytral epipleuron without a transverse carina at the humeral angle, 8) the prosternal process variable in shape (Figs 10–16), but extending to metaventrite, 9) the metatrochanter extremely large relative to the metafemur, approximately 0.6 × length of metafemur (Figs 17–23), 10) overall, the habitus elongate to oval, and the lateral pronotal and elytral margins nearly continuously and shallowly curved (Figs 1–9), and 11) the lateral lobes of the aedeagus two-segmented (Figs 24–30). In addition, a new character was identified based on new material with better-preserved male genitalia. Species of *Fontidessus* have a distinctive ventral sclerite associated with the male median lobe. In most species, this sclerite is short and oval (Figs 26B, 28–30B), but in *Fontidessus toboganensis* and *Fontidessus aquarupe* it is elongate (Figs 25B, 27B). In *Fontidessus microphthalmus*, though, the median lobe is complex with large dorsal and ventral portions (Fig. 24). The dorsal part may be homologous with the dorsal sclerite of other taxa. This species is different in several respects from other *Fontidessus*, and it is possible the ventral portion of the median lobe is, however, not homologous with the ventral sclerite in other taxa. *Fontidessus* is similar to *Spanglerodessus* Miller & García, but that genus lacks natatory setae (a possibly derived condition based on life in hygropetric habitats where swimming is not so important) and has a body that is very robust with the lateral pronotal margins extremely broadly expanded and rounded with the lateral margin of the body distinctly discontinuous between the elytron and pronotum. Also, the male median lobe of the single species, *Spanglerodessus shorti* Miller & García, does not have the distinctive ventral sclerite of *Fontidessus*. Nevertheless, the two genera appear to be similar.

#### Natural history.

**Figures 31–33.**
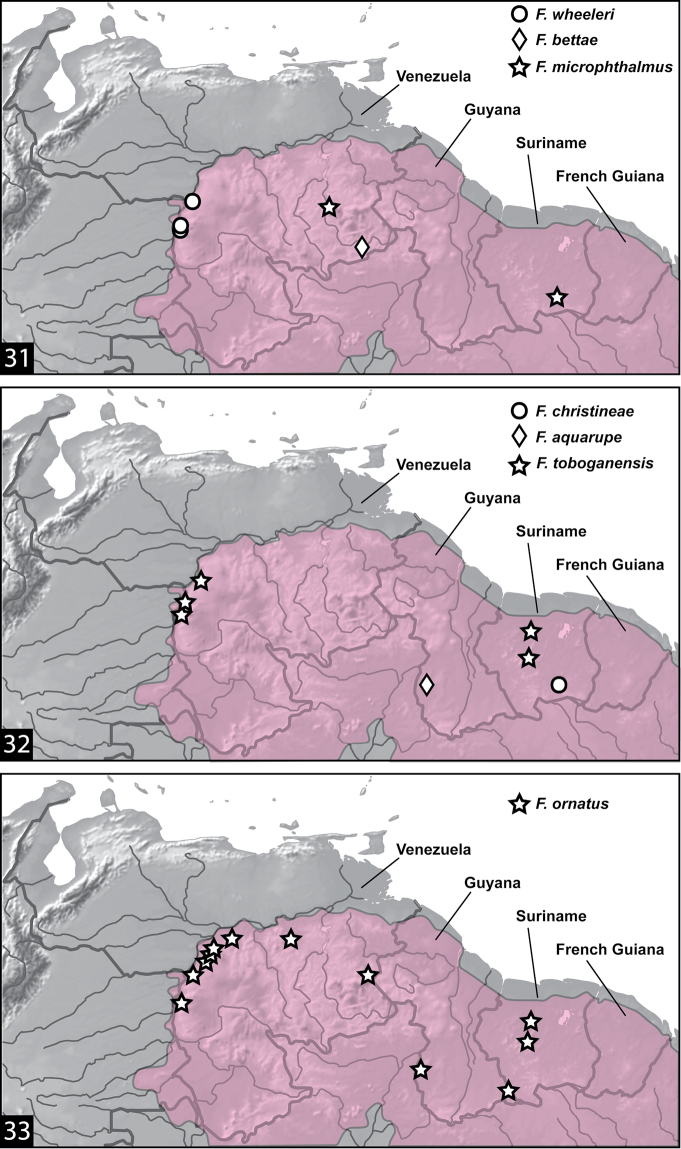
*Fontidessus* species, distributions. Pink area indicates Guiana Shield craton.

**Figures 34–36.**
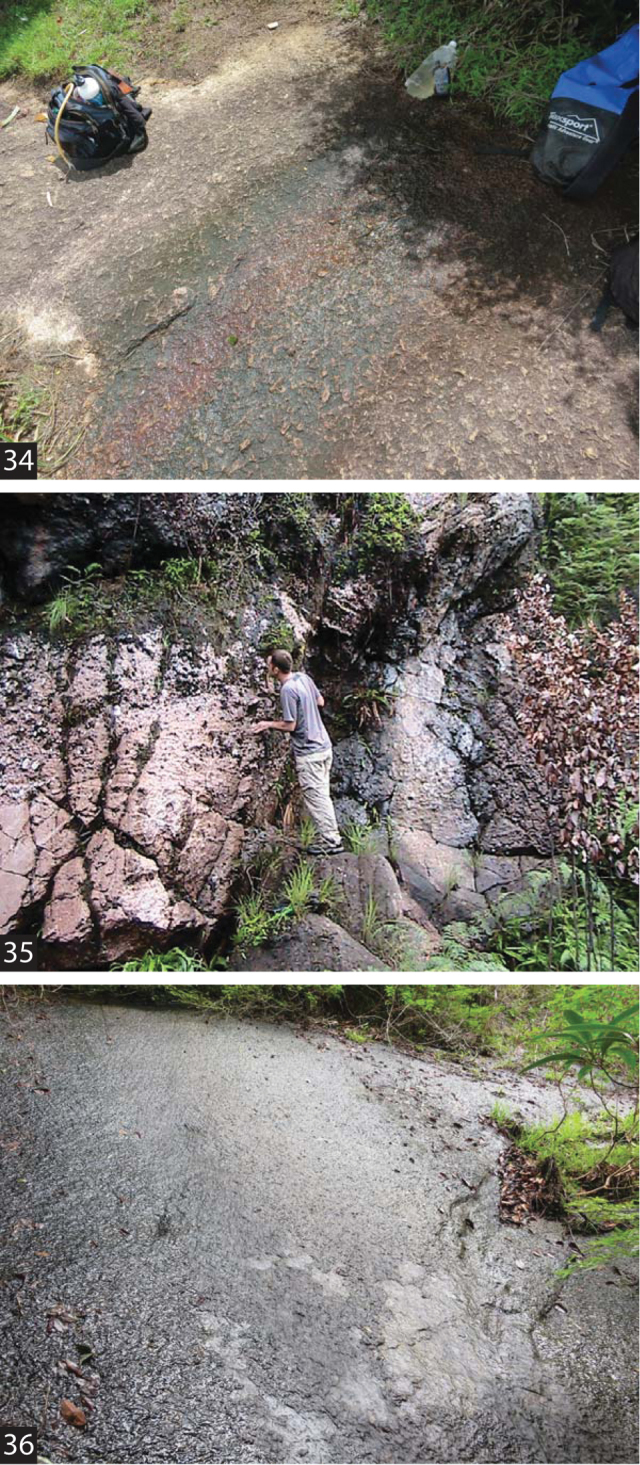
*Fontidessus* species, habitat. **34** collection site for paratypes of *Fontidessus microphthalmus* (SR12–0324–01A) **35** collection site for paratypes of *Fontidessus christineae* (SR12–0324–01C) **36** type locality for *Fontidessus bettae* (VZ10–0716–03B).

All the known species of *Fontidessus* are hygropetric (Figs 34–36), and most are known from only one or few localities. They are characteristic of marginal and small aquatic habitats on inselbergs or other rock surfaces (Figs 34, 36) and the margins of waterfalls (Fig. 35) in the Guaiana Shield region of northern South America (Figs 31–33). They can often be extremely abundant in these habitats. Several of the species co-occur, but others are the only known species at some sites (Figs 31–33).

#### Discussion.

Two previously described species, *Fontidessus ornatus* and *Fontidessus wheeleri*, were based on few specimens. Each of the male holotype specimens had been previously dissected, and it clearly appears, based on acquisition and examination of new material, that the male median lobes had been switched between the two and each median lobe suffered some damage during its dissection. Therefore, the incorrect male genitalia were associated with each species in the original descriptions, and the descriptions of these structures were based on somewhat damaged material (Miller and Spangler 2008). This is corrected in the following treatments.

#### Checklist of *Fontidessus* species

*Fontidessus* Miller & Spangler, 2008

*Fontidessus aquarupe* Miller & Montano, sp. n.: Guyana.

*Fontidessus bettae* Miller & Montano, sp. n.: Venezuela.

*Fontidessus christineae* Miller &Montano, sp. n.: Suriname.

*Fontidessus microphthalmus* Miller & Montano, sp. n.: Suriname, Venezuela.

*Fontidessus ornatus* Miller in Miller and Spangler 2008: Guyana, Suriname, Venezuela.

*Fontidessus toboganensis* Miller & Spangler, 2008: Suriname, Venezuela.

*Fontidessus wheeleri* Miller in Miller and Spangler 2008: Venezuela.

### Fontidessus microphthalmus

Taxon classificationAnimaliaColeopteraDytiscidae

Miller & Montano sp. n.

http://zoobank.org/40C0D37E-D76E-4F96-8051-A090E6A1DF91

Figs 1 , 10 , 17 , 24 , 31 , 34

#### Type locality.

Venezuela, Bolivar, Auyan–tepui, 5°51'N, 62°33'W.

#### Diagnosis.

*Fontidessus microphthalmus* is small (TL = 1.4–1.6 mm), but larger than all species except *Fontidessus toboganensis*, *Fontidessus bettae*, and *Fontidessus aquarupe* which are comparable in size (Figs 1–5). The dorsum is more pale in color than other species (darker along the elytral suture and lighter at the apex, but without distinctive maculae), and the eyes are relatively smaller than other species (Fig. 1, HW/EW = 1.4–1.5). The male genitalia are distinctive. The median lobe has two parts. The dorsal part is extremely broad, apically truncate and medially has a narrow emargination (Fig. 24A). The ventral part is elongate and apically pointed (Fig. 24A). The lateral lobes curve inward apically between the two parts of the median lobe (Fig. 24A).

#### Description.

*Measurements*. TL = 1.4–1.6 mm, GW = 0.8–0.9 mm, PW = 0.7–0.8 mm, HW = 0.5–0.6 mm, EW = 0.4 mm, TL/GW = 1.8, HW/EW = 1.4–1.5. Body (Fig. 1) broadly oval, dorsoventrally somewhat compressed; lateral outline nearly continuous between pronotum and elytron; lateral margins of pronotum gently curved; lateral margins of elytron evenly and gently curved.

*Coloration*. Head yellow brown, pronotum yellow, brown medially along posterior margin (Fig. 1); elytron yellow brown, apically lighter yellow brown. Ventral surfaces of thorax and abdomen brown except prosternum, prosternal process, propleuron and pronotal epipleuron yellow-brown; appendages yellow to yellow-brown.

*Sculpture and structure*. Head with very fine, inconspicuous, irregular punctation, surface between punctures shiny with distinct microreticulation; eyes small (Fig. 1, HW/EW = 1.4–1.5). Pronotal surface similar to that of head; with posterior angles obtuse; lateral bead narrow, of even width throughout, lateral margins weakly curved; pronotal striae finely incised, extending about 1/3 distance across pronotum (Fig. 1). Elytron with anterolateral angle obtuse, not extended anteriorly (Fig. 1); surface similar to pronotum, covered with fine microreticulation. Prosternal process narrow, lateral margins evenly curved and posteriorly convergent, apex of process narrowly rounded (Fig. 10); metacoxal process with lateral lobe minute. Pro- and mesotarsi relatively narrow in both male and female, but broader in male. Metatrochanter extremely large relative to metafemur (Fig. 17).

*Male genitalia*. Median lobe complex with two sections 1) an elongate ventral structure that is broad and evenly convergent to a narrowly rounded apex in dorsal aspect (Fig. 24A) and slender and evenly convergent to slight curved apex in lateral aspect (Fig. 24B), and 2) an elongate dorsal structure that is very broad, apically broadly truncate and medially narrowly emarginate in dorsal aspect (Fig. 24A) and slender in lateral aspect (Fig. 24B). Lateral lobes elongate, slender with apical segment medially curved between ventral and dorsal portions of median lobe (Fig. 24A).

#### Variation.

No significant variation was observed among the few specimens examined.

**Etymology.** The name of this species is derived from the Greek *micro*- meaning “small” and *ophthalmos* meaning “eye” for the relatively small eyes present in this species.

#### Distribution and habitat.

The species is known from two sites, one on the Auyan Tepui of the Gran Sabana of Venezuela, and the other on Kasikasima in the Sipilawini District of Suriname (Fig. 31) suggesting that the species may be characteristic of the Guyana Highlands. The specimens from Suriname are clearly hygropetric (Fig. 34), but the habitat is not known for the Venezuela specimens which were collected in an “intercept trap”.

#### Comments.

This is a problematic species. It is, in many ways, like other species of *Fontidessus*, but the male median lobe is unusually modified and the “ventral sclerite” is either absent or the large ventral part (Fig. 24) is homologous with that structure in the other species. Also, the eyes are quite small (Fig. 1). In some ways, it approaches *Spanglerodessus shorti* Miller and García in being broad, but its shape and structure, with the median lobe divided into two portions and the dorsal portion apically emarginate, is much more like species of *Fontidessus*.

#### Material examined.

Holotype in MIZA: ♂ labeled, “VENEZUELA. Bolivar Auyan–tepui 1700m 5°51'N, 62°33'W 7–14–94 J.L.Garcia–A.Chacón/ Trampa interceptacion [“intercept trap”]/ HOLOTYPE: *Fontidessus microphthalmus* Miller & Montano, 2014 [red label with black line border]”. Paratypes: 4 labeled same as holotype except “…PARATYPE: *Fontidessus microphthalmus* Miller & Montano, 2014 [blue label with black line border]”; 9 labeled “SURINAME: Sipaliwini District 2°58.555'N, 55°24.739'W 515 m Camp 4 (summit), Kasikasima leg. A. Short; small seepage on top of METS trail; 24.iii.2012 2012 CI–RAP Survey SR12–0324–01A/ PARATYPE: *Fontidessus microphthalmus* Miller & Montano, 2014 [blue label with black line border]”.

### Fontidessus aquarupe

Taxon classificationAnimaliaColeopteraDytiscidae

Miller & Montano sp. n.

http://zoobank.org/99100604-1FF8-4FC9-B864-4D5D2A544310

Figs 2 , 10 , 17 , 24 , 32

#### Type locality.

Guyana, Region IX, Kusad Mts, Mokoro Creek, 2°48.531'N, 59°51.900'W.

#### Diagnosis.

*Fontidessus aquarupe* is small (TL = 1.5–1.7 mm), but larger than all *Fontidessus* species except *Fontidessus toboganensis* and *Fontidessus bettae*, which are comparable in size (Figs 1–5). The dorsum is dark with diffuse yellow maculae on the pronotum and elytron. The male genitalia are distinctive. The median lobe is broad and apically broadly pointed and curved dorsad (Fig. 24A, C). The ventral sclerite is long and broad, and apically subtruncate with distinct lateral pointed lobes (Fig. 24B). The lateral lobes are basally broad and apically are elongate and slender (Fig. 25A, D). The prosternal process is relatively broad, flattened and apically narrowly rounded (Fig. 11).

#### Description.

*Measurements*. TL = 1.5–1.7 mm, GW = 0.9–1.0 mm, PW = 0.8–0.9 mm, HW = 0.5–0.6 mm, EW = 0.3-0.4 mm, TL/GW = 1.7, HW/EW = 1.5–1.6. Body (Fig. 2) oval, robust, dorsoventrally somewhat compressed; lateral outline nearly continuous between pronotum and elytron; lateral margins of pronotum evenly curved; lateral margins of elytron evenly and broadly curved.

*Coloration*. Head dark brown; pronotum dark brown, black along anterior and posterior margins, with yellow transverse macula on each side anterior to middle (Fig. 2); elytron brown with diffuse pale areas anteriorly and with posterior extensions along lateral margins and near suture, apex with triangular pale area (Fig. 2). Ventral surfaces black to dark red-brown except prothorax, including prosternal process, yellow; antennae and appendages yellow to yellow-brown.

*Sculpture and structure*. Head with very fine, inconspicuous, irregular punctation, surface between punctures shiny with distinct microreticulation comprised of minute, isodiametric cells; eyes medium in size (Fig. 2, HW/EW = 1.5–1.6). Pronotal surface similar to head; with posterior angles obtuse; lateral bead narrow, of even width throughout, lateral margins weakly curved; pronotal striae fine, extending nearly 1/2 distance across pronotum (Fig. 2). Elytron with anterolateral angle obtuse, not extended anteriorly (Fig. 2); surface similar to pronotum, covered with fine microreticulation. Prosternal process moderately narrow, flattened, lateral margins posteriorly convergent, apex of process narrowly rounded (Fig. 11); metacoxal process with lateral lobe minute. Pro- and mesotarsi relatively narrow in both male and female, but slightly broader in male. Metatrochanter extremely large relative to metafemur (Fig. 18).

*Male genitalia*. Median lobe in dorsal aspect elongate, broad, apically abruptly constricted, terminating in two elongate, narrow rami with medial deep, narrow emargination, apex with inconspicuous, diaphanous lobes laterally (Fig. 25A); in lateral aspect slender, elongate, conspicuously recurved with apical portion very slender and apically narrowly pointed (Fig. 25C); with elongate ventral sclerite that is broad and apically truncate, terminating in pointed lobes laterally (Fig. 25B). Lateral lobes elongate, apically abruptly sinuate, basally broad (Fig. 25D).

#### Variation.

Specimens vary in the extent of dorsal coloration, particularly the extent to which the anterior diffuse elytral macula extends posteriorly along the lateral and sutural margins.

#### Etymology.

This species is named *aquarupe* from the Latin, *aqua*, meaning “water”, and *rupe*, meaning “rock” or “cliff” since specimens are known from hygropetric habitats.

#### Distribution and habitat.

*Fontidessus aquarupe* is known only from seepages and wet rocks near a single site in the Kusad Mountains of southwestern Guaya (Fig. 32).

#### Material examined.

Holotype in CSBD: male labeled, “GUYANA: Regions IX 2°48.531'N, 59°51.900'W, 170m Kusad Mts., large seepage nr. Basecamp: on wet rocks leg. A. Short & W. Washington GY13-1024-03C/ SEMC1047996 KUNHM-ENT [barcode label]/ HOLOTYPE: *Fontidessus aquarupe* Miller & Montano, 2014 [red label with black line border]”. Paratypes: 69 labeled same as holotype except “…/PARATYPE: *Fontidessus aquarupe* Miller & Montano, 2014 [blue label with black line border]”; 69 labeled, “GUYANA: Region IX 2°48.531'N, 59°51.900'W 170m Kusad Mts., Mokoro Creek main seepage area leg. Short, Isaacs, Salisbury 27.x.2013; GY13-1027-03B /PARATYPE: *Fontidessus aquarupe* Miller & Montano, 2014 [blue label with black line border]”.

### Fontidessus bettae

Taxon classificationAnimaliaColeopteraDytiscidae

Miller & Montano sp. n.

http://zoobank.org/6D39FB62-9AC2-4DC5-B43A-C2001DCCD350

Figs 3 , 12 , 19 , 26 , 31 , 35

#### Type locality.

Venezuela, Bolivar State, Gran Sabana, E Pauji, Salto Catedral, 4°31'19.1"N, 61°31'34.0"W.

#### Diagnosis.

*Fontidessus bettae* is small (TL = 1.4 mm), but larger than all species except *Fontidessus toboganensis*, *Fontidessus aquarupe*, and *Fontidessus microphthalmus*, which are comparable in size (Figs 1–5). The dorsal maculae are distinctive. The elytron has a transverse, well-demarcated yellow macula near the anterior margin and a smaller, subapical yellow macula (Fig. 3). The anterior macula does not have medial or lateral posterior extensions along the disc (Fig. 3) as is often the case in *Fontidessus tambopaticus* (Fig. 5). The eyes in *Fontidessus bettae* are larger (Fig. 3, HW/EW = 1.5–1.6) than in *Fontidessus microphthalmus* (Fig. 1, HW/EW = 1.4–1.5). The male median lobe terminates in a narrow, pointed apex with only small lateral hyaline lobes (Fig. 26).

#### Description.

*Measurements*. TL = 1.4–1.5 mm, GW = 0.9 mm, PW = 0.7 mm, HW = 0.4–0.5 mm, EW = 0.3 mm, TL/GW = 1.7–1.8, HW/EW = 1.5–1.6. Body (Fig. 3) broadly oval; lateral outline nearly continuous between pronotum and elytron; lateral margins of pronotum gently curved; lateral margins of elytron evenly and gently curved.

*Coloration*. Head brown, pronotum dark brown along anterior and posterior margins with prominent yellow transverse region near anterior margin, darker yellow brown medially (Fig. 3); elytron brown with distinct, well-defined, yellow, transverse but slightly oblique macula near base and yellow triangular shaped macula near apex (Fig. 3). Ventral surfaces of thorax and abdomen dark brown to black except prosternum, prosternal process, propleuron and pronotal epipleuron yellow-brown; appendages yellow to yellow-brown.

*Sculpture and structure*. Head with very fine, inconspicuous, irregular punctation, surface between punctures shiny with distinct microsculpture in the form of small cells; eyes medium in size (Fig. 3, HW/EW = 1.5–1.6). Pronotal surface similar to that of head; with posterior angles obtuse; lateral bead narrow, of even width throughout; pronotal striae finely incised, extending nearly 1/2 distance across pronotum (Fig. 3). Elytron with anterolateral angle obtuse, not extended anteriorly (Fig. 3); surface similar to pronotum. Prosternal process moderately broad, lateral margins evenly curved, apex of process narrowly rounded (Fig. 12); metacoxae impunctate, but with distinct microreticulation; metacoxal process with lateral lobe minute. Pro- and mesotarsi relatively narrow in both male and female, but slightly broader in male. Metatrochanter very large in relation to metafemur (Fig. 19).

*Male genitalia*. Median lobe in dorsal aspect broad basally, with lateral margins approximately evenly tapered to extremely slender, bifid apex, with small, but distinctive, hyaline lobes laterally (Fig. 26A); in lateral aspect moderately broad basally, apical portion moderately straight, lateral margins evenly tapered to extremely slender apex (Fig. 26C); with broad, rounded dorsal sclerite (Fig. 26B). Lateral lobe in lateral aspect broad basally, apical segment slender and moderately straight (Figs 26D).

#### Variation.

There is some minor variation in the extent and distinctiveness of the dorsal maculae, but relatively little variation among the few specimens examined.

#### Etymology.

This species is named to honor the second author’s grandmother, Elizabeth (Betty) Baca, who deeply inspired her to live well and seek knowledge.

#### Distribution and habitat.

*Fontidessus bettae* is known only from the type series in the Gran Sabana of Venezuela (Fig. 31). It was collected from “waterfall seeps”, a hygropetric habitat along the margin of a waterfall (Fig. 35).

#### Material examined.

Holotype in MIZA: male labeled, “VENEZUELA: Bolivar State 4°31'19.1"N, 61°31'34.0"W, 860 m Gran Sabana, E Pauji. Salto Catedral 16.vii.2010 leg. Short & Tellez waterfall seeps; VZ10–0716–03B/ HOLOTYPE: *Fontidessus bettae* Miller & Montano, 2014 [red label with black line border]”. Paratypes: 19 labeled same as holotype except “…/PARATYPE: *Fontidessus bettae* Miller & Montano, 2014 [blue label with black line border]”.

### Fontidessus toboganensis

Taxon classificationAnimaliaColeopteraDytiscidae

Miller & Spangler, 2008

Figs 4 , 5 , 13 , 20 , 27 , 32

Fontidessus toboganensis Miller & Spangler, 2008: 48.

#### Type locality.

Venezuela, Territorio Federal Amazonas, 40km S Puerto Ayacucho, at El Tobogan, Coromoto.

#### Diagnosis.

Although small for diving beetles, this species is relatively large for the genus (Figs 4, 5, TL = 1.5–1.6 mm). The prosternal process is moderately broad and parallel-sided and has the apex broadly truncate (Fig. 13). The male genitalia are distinctive with the median lobe elongate, slender and broadly recurved in lateral aspect (Fig. 27C). There is an elongate ventral sclerite that fits into the ventral groove of the median lobe (Figs 27B, C). Specimens are variable in coloration with specimens from Suriname indistinctly marked with maculae (Fig. 4) and specimens from Venezuela marked with diffuse but distinctive maculae on the elytra (Fig. 5).

#### Redescription.

*Measurements*. TL = 1.5–1.6 mm, GW = 0.8–0.9 mm, PW = 0.6–0.7 mm, HW = 0.4–0.5 mm, EW = 0.3–0.4 mm, TL/GW = 1.8–1.7, HW/EW = 1.6–1.7. Body (Figs 4, 5) oval, elongate; lateral outline nearly continuous between pronotum and elytron; lateral margins of pronotum gently curved; lateral margins of elytron evenly and gently curved.

*Coloration*. Head yellow brown, pronotum yellow brown, brown medially along posterior margin; elytron brown with the following yellow maculae: 1) a basal macula in a band extending from lateral to sutural margins, posterior margin of macula irregular, often with medial extension posteriorly along elytral suture, 2) a lateral longitudinal macula at humeral angle, 3) a narrow longitudinal macula along lateral margin medially, and 4) a subtriangular subapical macula (Figs 4, 5). Many specimens with elytral maculae reduced or indistinct (Fig. 4). Ventral surfaces of thorax and abdomen brown except prosternum, prosternal process, propleuron and pronotal epipleuron yellow-brown; appendages yellow to yellow-brown.

*Sculpture and structure*. Head with very fine, inconspicuous, irregular punctation, surface between punctures shiny with indistinct microsculpture in the form of small cells; eyes medium in size (Figs 4, 5, HW/EW = 1.6–1.7). Pronotal surface similar to that of head; with posterior angles obtuse; lateral bead narrow, of even width throughout; pronotal striae finely incised, extending nearly 1/2 distance across pronotum (Figs 4, 5). Elytron with anterolateral angle obtuse, not extended anteriorly (Figs 4, 5); surface similar to pronotum. Prosternal process broad, lateral margins subparallel, apex of process broadly rounded (Fig. 13); metacoxal process with lateral lobe minute. Pro- and mesotarsi relatively narrow in both male and female, but slightly broader in male. Metatrochanter very large relative to metafemur (Fig. 20).

*Male genitalia*. Median lobe in dorsal aspect elongate, slender, apically terminating in two elongate, narrow rami with medial deep, narrow emargination, long apicies distinctly expanded laterally (Fig. 27A); in lateral aspect slender, elongate, conspicuously recurved with apical portion very slender and apically narrowly pointed (Fig. 27C); with elongate ventral sclerite that is pointed apically (Fig. 27B). Lateral lobes elongate, apically slightly curved and narrowly rounded (Fig. 27D).

*Female genitalia*. Bursa copulatrix elongate and narrow; spermathecal duct long, slender, somewhat coiled, expanded before receptacle; receptacle small, about half size of spermatheca, intermediate duct between receptacle and spermatheca broad; spermatheca globular, spermathecal spine prominent, but not elongate; fertilization duct elongate, tightly curved in spiral, attaching to large fertilization sac; vagina elongate, moderately narrow; gonocoxae elongate, slender with abrupt, small, triangular expansion at apex.

#### Variation.

This species exhibits considerable variability in the extent of yellow coloration on the dorsal surface. Specimens from Venezuela are usually heavily marked with yellow maculae and fasciae on the elytron and have the pronotum and head yellow (Fig. 5). Specimens from Suriname, in contrast, have the elytral maculae reduced and very indistinct and the head and pronotum darker (Fig. 4).

#### Distribution and habitat.

*Fontidessus toboganensis* is known from the extreme western margin of the Guayana Shield in the area around Puerto Ayacucho, Venezuela and from a few localities in Suriname (Fig. 32). In both areas it is extremely common and abundant in hygropetric habitats. The broadly disjunct distribution and differences in coloration suggest the possibility that the two populations are different species, but the male genitalia are shaped the same and the body is similarly shaped and sized. This is similar to *Fontidessus ornatus* which is also darker and less maculate in eastern localities than in western ones, though that species is also known from localities in between.

#### Material examined.

~627 specimens from the following localities: **VENEZUELA:** Amazonas: 40km S Puerto Ayacucho, El Tobogan, Caño Coromoto 5.38678°N, 67.61537°W; nr Iboruwa: Tobogancito, 5.8069°N, 67.43855°W; Bolivar: Los Pijiguaos at rock outcrop, 6.59361°N, 66.82063°W. **SURINAME:** Sipaliwini District, Raleighfallen Nature Reserve plateau below Voltzberg, seepage, 4.68276°N, 56.1877°W. Sipaliwini District, Tafelberg Summit, nr Augustus Creek Camp, large seepage area, 3°55.600'N, 56°11.300'W 600m; Sipaliwini District, Tafelberg Summit, near Caiman Creek Camp, washing seepage, 3°53.942'N, 56°10.849'W, 733m.

### Fontidessus christineae

Taxon classificationAnimaliaColeopteraDytiscidae

Miller & Montano sp. n.

http://zoobank.org/84A7F7E3-E51D-4F5C-BA12-5C3E87F9A5CC

Figs 6 , 14 , 21 , 24 , 28 , 32 , 36

#### Type locality.

Suriname, Sipaliwini District, Kasikasima, small seepage on top of METS trail, 2°58.555'N, 56°24.739'W, 515m.

#### Diagnosis.

*Fontidessus christineae* is extremely small (<1.2 mm) and very dark testaceous dorsally with only some diffuse, transverse yellow margins on the pronotum (Fig. 6). The male median lobe terminates apically in two broadly lobate rami (Fig. 28).

#### Description.

*Measurements*. TL = 1.1–1.2 mm, GW = 0.7 mm, PW = 0.6 mm, HW = 0.4 mm, EW = 0.3 mm, TL/GW = 1.7, HW/EW = 1.5–1.6. Body (Fig. 6) broadly oval; lateral outline nearly continuous between pronotum and elytron; lateral margins of pronotum gently curved; lateral margins of elytron evenly and gently curved.

*Coloration*. Head dark red-brown, lighter anteriorly, pronotum dark red-brown along anterior and posterior margins with indistinct yellow-red transverse region across anterior half, darker yellow brown medially (Fig. 6); elytron dark red-brown (Fig. 6). Ventral surfaces of thorax and abdomen dark brown to dark red-brown except prosternum, prosternal process, propleuron and pronotal epipleuron red-brown; appendages yellow to yellow-brown.

*Sculpture and structure*. Head with very fine, inconspicuous, irregular punctation, surface between punctures shiny with distinct microsculpture in the form of small cells; eyes medium in size (Fig. 6, HW/EW = 1.5–1.6). Pronotal surface similar to that of head; with posterior angles obtuse; lateral bead narrow, of even width throughout; pronotal striae finely incised, extending nearly 1/2 distance across pronotum (Fig. 6). Elytron with anterolateral angle obtuse, not extended anteriorly (Fig. 6); surface similar to pronotum but with fine microsculpture cells more distinct. Prosternal process moderately broad, lateral margins slightly angulate, apex of process narrowly rounded (Fig. 14); metacoxae impunctate, but with distinct microreticulation; metacoxal process with lateral lobe minute. Pro- and mesotarsi broader in male than female. Metatrochanter very large relative to metafemur (Fig. 21).

*Male genitalia*. Median lobe in dorsal aspect deeply emarginate medially, each arm moderately slender, lateral margin sinuate, medial margin evenly curved, apex expanded and directed medially, with large, hyaline, rounded lobes laterally (Fig. 28A); in lateral aspect very broad, medially curved ventrad, apex tapered to sub-truncate apex (Fig. 28C); with broad, rounded dorsal sclerite (Fig. 28B). Lateral lobe in lateral aspect with basal segment broad, dorsally acute along margin, apical segment slender, apically slightly expanded to narrowly rounded apex, with fine setae along ventral margin (Figs 28D).

#### Variation.

No significant variation was identified in the single series of this species.

#### Etymology.

This species is named after the second author’s mother, Christine Anne Montano, who taught her to work hard and seek to achieve.

#### Distribution and habitat.

*Fontidessus christineae* is known only from one area, on Mount Kasikasima in Suriname (Fig. 32) in a “small seepage” and in the “main seepage area” (Fig. 36). The localities are at 400 and 515 meters

#### Material examined.

Holotype in NZCS male labeled, “SURINAME: Sipaliwini District 2°58.555'N, 56°24.739'W, 515 m Camp 4 (summit), Kasikasima leg. A. Short; small seepage on top of METS trail; 24.iii.2012 2012 CI–RAP Survey SR12–0324–01A/ SEMC1087097 KUNHM–ENT [barcode label]/ HOLOTYPE *Fontidessus christineae* Miller, 2013 [red label with black line border]”. Paratypes 7, 6 labeled same as holotype except with KUNHM–ENT numbers SEMC1087380, SEMC1087091, SEMC1087089, SEMC1087088, SEMC1087096, SEMC1087098. One paratype labeled “SURINAME: Sipaliwini District 2°58.613'N, 55°24.683'W, 400 m Camp 4 (high), Kasikasima leg. A. Short; main seepage area 24.iii.2012; SR12–0324–01C 2012 CI–RAP Survey/ SEMC1088139 KUNHM–ENT”. Paratypes also with “…/PARATYPE *Fontidessus christineae* Miller, 2013 [blue label with black line border]”.

### Fontidessus ornatus

Taxon classificationAnimaliaColeopteraDytiscidae

Miller, 2008

Figs 7 , 8 , 15 , 22 , 29 , 33

Fontidessus ornatus Miller, 2008: 50 in Miller and Spangler 2008.

#### Type locality.

Venezuela, Territorio Federal Amazonas, 40km S Puerto Ayacucho, at El Tobogan, Coromoto.

#### Diagnosis.

*Fontidessus ornatus* are very small (Figs 7, 8, TL = 1.09–1.20). Specimens are diffusely marked with yellow on the elytron anteriorly, medially and laterally (Fig. 8) or are nearly entirely darkly colored (Fig. 7). The median lobe terminates apically in two slender rami with two very broad, lateral hyaline lobes (Fig. 29).

#### Redescription.

*Measurements*. TL = 1.1–1.2 mm, GW = 0.6–0.7 mm, PW = 0.6–0.7 mm, HW = 0.4–0.5 mm, EW = 0.1–0.2 mm, TL/GW = 1.8–1.9, HW/EW = 1.5–1.6. Body (Figs 7, 8) oval, elongate; lateral outline slightly continuous between pronotum and elytron; lateral margins of pronotum gently curved; lateral margins of elytron evenly and gently curved.

*Coloration*. Head yellow brown, darker in region near medial margins of eyes; pronotum yellow brown, diffusely brown medially along posterior margin and in narrow band along anterior margin; elytron brown with diffuse yellow pattern, yellow basally, laterally along about ¾ length of elytra and in subtriangular subapical area (Fig. 8). Other specimens much darker, with elytral maculae reduced overall (Fig. 7). Ventral surfaces of thorax and abdomen brown except prosternum, prosternal process, propleuron and pronotal epipleuron yellow-brown; appendages yellow to yellow-brown.

*Sculpture and structure*. Head with very fine, inconspicuous, irregular punctation, surface between punctures shiny with indistinct microsculpture in the form of small cells; eyes medium in size (Figs 7, 8, HW/EW = 1.5–1.6). Pronotal surface similar to that of head; with posterior angles obtuse; lateral bead slender, of even width throughout; pronotal striae finely incised, extending nearly 1/2 distance across pronotum (Figs 7, 8). Elytron with anterolateral angle obtuse, not extended anteriorly (Figs 7, 8); surface similar to pronotum but with microsculpture more indistinct. Prosternal process relatively narrow, lateral margins tapered to narrowly rounded apex (Fig. 15); metacoxal process with lateral lobe minute. Pro- and mesotarsi relatively narrow in both male and female. Metatrochanter very large relative to metafemur (Fig. 22).

*Male genitalia*. Median lobe in dorsal aspect complex, apically terminating in two elongate, narrow rami with medial deep, narrow emargination, laterally with broad, hyaline lobes (Fig. 29A); in lateral aspect narrow, straight, apically with distinct subapical hyaline lobe and apex slightly bent, apically very slender (Fig. 29C); ventral sclerite short, broad, apically rounded (Fig. 29B). Lateral lobe slender medially, apically expanded and broadly rounded (Fig. 29D).

#### Variation.

This species exhibits considerable variation in color between the two main regions in which it has been collected (Surinam/Guyana and Venezuela). The eastern specimens are noticeably darker in overall coloration (Fig. 7) than those from Venezuela which are more distinctly marked with yellow maculae on the elytra and lighter in color on the pronotum and head (Fig. 8). The distinctive male genitalia (Fig. 29) are similar in each of the two populations, though, and so this color variation is regarded as intraspecific.

#### Distribution and habitat.

*Fontidessus ornatus* is more widespread and common than other species in the genus. It ranges from the western margin of the Guiana Shield through northeastern Venezuela to central and southern Suriname (Fig. 33). It is found in hygropetric habitats.

#### Comments.

The original description of *Fontidessus ornatus* included 15 specimens. This type series was mixed together with specimens of *Fontidessus toboganensis* and *Fontidessus wheeleri*. The holotype of *Fontidessus ornatus* had already been dissected, but based on considerable new material, it appears that the genitalia had been damaged somewhat and, worse yet, exchanged with the genitalia of what became the holotype of *Fontidessus wheeleri*. The genitalia are redescribed and reillustrated here based on newly dissected specimens with greater precision and in correct association with the species to which they belong.

#### Material examined.

~400 total from the following localities: **VENEZUELA:** Amazonas: 40km S Puerto Ayacucho, El Tobogan, Caño Coromoto 5.38678°N, 67.61537°W; Bolivar: Los Pijiguaos at rock outcrop, 6.59361°N, 66.82063°W; rock outcrop by Rio Cuchivero 7.45835°N, 65.86821°W; outcrop ca 15km NE Pijiguaos, 6.96506°N, 66.60653°W; rock outcrop by morichal, 6.86993°N, 66.59083°W; 2km E Rio Chucivero, 7.45835°N, 65.86821°W; ca 25km E El Burro, 6.21794°N, 67.24066°W; along La Escalera, Hwy 10, 6.08186°N, 61.39797°W; ca 14km E Rio Aro, 7.23988°N, 64.0322°W. **SURINAME:** Sipaliwini District: camp 2, Sipaliwini Riv., 2.18288°N, 56.78725°W; Raleighfallen Nature Reserve plateau below Voltzberg, 4.68276°N, 56.1877°W; Sipaliwini District: Tafelberg Summit, nr Augustus Creek Camp, large seepage area, 3°55.600'N, 56°11.300'W 600m. **GUYANA:** Region IX, Kusad Mtns, large seepage nr. Basecamp; on wet rocks, and Mokoro Creek main seepage area, 2°48.531'N, 59°51.900'W, 170m.

### Fontidessus wheeleri

Taxon classificationAnimaliaColeopteraDytiscidae

Miller, 2008

Figs 9 , 16 , 23 , 30 , 31

Fontidessus wheeleri Miller, 2008 in Miller and Spangler 2008: 50.

#### Type locality.

Venezuela, Territorio Federal Amazonas, 40km S Puerto Ayacucho, at El Tobogan, Coromoto.

#### Diagnosis.

*Fontidessus wheeleri* are small (Fig. 9, TL = 1.2–1.3 mm). Specimens are marked on the elytra with transverse and longitudinal yellow maculae that are distinctly demarcated. The male median lobe terminates in two, short, slender rami with small, subapical, lateral, hyaline lobes (Fig. 30).

#### Redescription.

*Measurements*. TL = 1.2–1.3 mm, GW = 0.6–0.7 mm, PW = 0.5–0.6 mm, HW = 0.4–0.5 mm, EW = 0.2–0.3 mm, TL/GW = 1.7–1.8, HW/EW = 1.6–1.7. Body (Fig. 9) oval, elongate; lateral outline slightly continuous between pronotum and elytron; lateral margins of pronotum gently curved; lateral margins of elytron evenly and gently curved.

*Coloration*. Head yellow brown, brown along posterior margins and medial margins of eyes; pronotum broadly brown along posterior margin, narrowly dark brown along anterior margin; elytron brown with the following yellow maculae: 1) a basal macula in an irregular band extending posteriorly along suture at medial margin, 2) a lateral longitudinal macula posterior to humeral angle, broader anteriorly and extending posteriorly in narrow band to near apex, 3) a subtriangular subapical macula (Fig. 9). Ventral surfaces of thorax and abdomen dark brown except prosternum, prosternal process, propleuron and pronotal epipleuron yellow-brown; appendages yellow to yellow-brown.

*Sculpture and structure*. Head with very fine, inconspicuous, irregular punctation, surface between punctures shiny, without distinct microsculpture; eyes medium in size (Fig. 9, HW/EW = 1.6–1.7). Pronotal surface similar to that of head, with posterior surface with fine microsculpture; with posterior angles obtuse; lateral bead slender, of even width throughout; pronotal striae finely incised, extending nearly 1/2 distance across pronotum (Fig. 9). Elytron with anterolateral angle obtuse, not extended anteriorly (Fig. 9); surface similar to pronotum, with microsculpture indistinct. Prosternal process elongate and slender, lateral margins slightly concave, apex of process rounded (Fig. 16); metacoxal process with lateral lobe small. Pro- and mesotarsi relatively narrow in both male and female, but slightly broader in male. Metatrochanter very large relative to metafemur (Fig. 23).

*Male genitalia*. Median lobe in ventral aspect broad, tapering slightly, apically bifid and slender, deeply and narrowly emarginate medially, with short, hyaline, sub-triangular lobes subapically on each side (Fig. 30A); in lateral aspect moderately broad, curved, with apical portion evenly tapered to pointed apex (Fig. 30C). Lateral lobe elongate, expanded apically, broadly rounded at apex, with series of long setae along dorsal margin (Fig. 30B).

#### Variation.

The extent of the dorsal maculae varies among specimens with some having the maculae reduced, but most with extensive maculae that are well-defined.

#### Distribution and habitat.

This species is known only from a few localities along the far western margin of the Guiana Shield around Puerto Ayacucho (Fig. 31). Specimens have been found in hygropetric situations.

#### Comments.

This species was originally based on two specimens (the holotype and a paratype) that were originally collected together with the type series of *Fontidessus ornatus* and *Fontidessus toboganensis*, which were described at the same time (Miller and Spangler 2008). The holotype of *Fontidessus wheeleri* had been dissected some time prior to the original description, but, based on a few additional specimens examined for this paper, the male genitalia were apparently somewhat damaged (torn and crushed somewhat), and also switched with those of the holotype of *Fontidessus ornatus*, which had also been dissected previously. For that reason, the male genitalia are redescribed and reillustrated here based on newly dissected specimens in association with the correct species based on examination on a few additional specimens. This species has been only rarely collected.

#### Material examined.

Six total specimens from the following localities: **VENEZUELA:** Amazonas: 40km S Puerto Ayacucho, El Tobogan, Caño Coromoto 5.38678°N, 67.61537°W; ca 15km S Puerto Ayacucho, 5.51038°N, 67.60181°W; Bolivar: ca 25km E El Burro, 6.21794°N, 67.24066°W.

### Key to the species of *Fontidessus*

**Table:** 

1	Size larger (Figs 1–5, TL > 1.4 mm)	2
–	Size smaller (Figs 6–9, TL < 1.3 mm	5
2	Eyes relatively smaller (Fig. 1, HW/EW < 1.5); male median lobe complex, apically with two parts, ventral part narrow, apically narrowly rounded, dorsal part broad, apically truncate and with medial emargination (Fig. 24)	*Fontidessus microphthalmus* sp. n.
–	Eyes relatively larger (HW/EW > 1.5); male median not comprised of two long, equally well-developed parts, instead comprised of a single lobe and a shorter, articulated ventral sclerite	3
3	Prosternal process apically subtruncate, broad (Fig. 13); ventral sclerite of median lobe elongate, slender, main portion of median lobe ending in slightly bifid, but laterally expanded and narrowly rounded apex (Fig. 27)	*Fontidessus toboganensis* Miller & Spangler
–	Prosternal process apically pointed; ventral sclerite various, either short and oval in shape, or elongate but broad and apically expanded laterally, but not elongate and slender	4
4	Elytron with diffuse basal macula, margins irregular and not well demarcated, often with posterior extensions (Fig. 2); ventral sclerite of median lobe elongated, but broad, apically laterally expanded into pointed lobes, main portion of median lobe broad, apically broadly pointed (Fig. 25)	*Fontidessus aquarupe* sp. n.
–	Elytron with distinctive basal macula which is obliquely transverse and has well demarcated and regular margins, without lateral or medial posterior extensions of the macula (Fig. 3); ventral sclerite of median lobe with oval, main portion of median lobe ending in slightly bifid, pointed apex (Fig. 26)	*Fontidessus bettae* sp. n.
5	Size extremely small (Fig. 6, TL < 1.2 mm); dorsal coloration dark testaceous with only diffuse, transverse yellow margins present on pronotum (Fig. 6); male median lobe apically terminating in two broadly lobate rami (Fig. 28)	*Fontidessus christineae* sp. n.
–	Size small, but usually larger (TL > 1.1 mm); dorsal coloration conspicuously marked with distinctive maculae on pronotum and elytra, or, if dorsally mostly darkly-colored, with at least pale, diffuse maculae evident apically and basally on elytron; male median lobe apically terminating in two slender rami	6
6	Prosternal process moderately elongate, lateral margins apically evenly tapered to pointed apex (Fig. 15); elytral maculations pale brown-yellow, diffuse and indistinctly demarcated (Figs 7, 8); male median lobe with large, hyaline, rounded lobes on each side (Fig. 29)	*Fontidessus ornatus* Miller
–	Prosternal process elongate, lateral margins apically slightly concave to sharply pointed apex (Fig. 16); elytral maculations bright yellow, distinctly demarcated (Fig. 9); male median lobe with small, hyaline, rounded lobes on each side (Fig. 30)	*Fontidessus wheeleri* Miller

## Supplementary Material

XML Treatment for Fontidessus

XML Treatment for Fontidessus microphthalmus

XML Treatment for Fontidessus aquarupe

XML Treatment for Fontidessus bettae

XML Treatment for Fontidessus toboganensis

XML Treatment for Fontidessus christineae

XML Treatment for Fontidessus ornatus

XML Treatment for Fontidessus wheeleri
